# Systematic dissection of dysregulated transcription factor–miRNA feed-forward loops across tumor types

**DOI:** 10.1093/bib/bbv107

**Published:** 2015-12-09

**Authors:** Wei Jiang, Ramkrishna Mitra, Chen-Ching Lin, Quan Wang, Feixiong Cheng, Zhongming Zhao

**Keywords:** pan-cancer, feed-forward loop, TF and miRNA regulatory network, gene and miRNA expression, drug repositioning

## Abstract

Transcription factor and microRNA (miRNA) can mutually regulate each other and jointly regulate their shared target genes to form feed-forward loops (FFLs). While there are many studies of dysregulated FFLs in a specific cancer, a systematic investigation of dysregulated FFLs across multiple tumor types (pan-cancer FFLs) has not been performed yet. In this study, using The Cancer Genome Atlas data, we identified 26 pan-cancer FFLs, which were dysregulated in at least five tumor types. These pan-cancer FFLs could communicate with each other and form functionally consistent subnetworks, such as epithelial to mesenchymal transition-related subnetwork. Many proteins and miRNAs in each subnetwork belong to the same protein and miRNA family, respectively. Importantly, cancer-associated genes and drug targets were enriched in these pan-cancer FFLs, in which the genes and miRNAs also tended to be hubs and bottlenecks. Finally, we identified potential anticancer indications for existing drugs with novel mechanism of action. Collectively, this study highlights the potential of pan-cancer FFLs as a novel paradigm in elucidating pathogenesis of cancer and developing anticancer drugs.

## Introduction

Transcription factors (TFs) are proteins that control the rate of transcription from DNA to messenger RNA (mRNA) through binding to specific DNA sequences [1]. A larger fraction of oncogenes and tumor suppressor genes encode TFs [2]. Aberrant TF activity can lead to dysregulation of genes involved in almost all known cellular processes related to tumorigenesis, such as apoptosis, proliferation, angiogenesis and metastasis [2, 3]. microRNAs (miRNAs) are small (∼22 nucleotides) noncoding RNAs that mainly repress gene expression at the posttranscriptional level by binding to the 3′ untranslated regions of the target mRNA [4]. Accumulating evidence has shown that miRNAs may play oncogenic and/or tumor-suppressive roles in a variety of human cancers [5]. To date, there were many Web servers and databases to predict TF targets, such as JASPAR [6] and TRANSFAC [7], or miRNA targets, such as TargetScan [8] and miRanda [9]. However, the predicted targets usually had high false-positive rates. With the accumulation of experimentally validated TF and miRNA regulations, several databases had been developed to collect these curated regulations, such as TRANSFAC [7] and TarBase [10].

Importantly, TFs and miRNAs can regulate the shared target genes in a coordinated fashion [11], thus, forming the feed-forward loops (FFLs) as regulatory units, in which TF regulates miRNA or miRNA regulates TF, and both of them co-regulate target genes [12]. According to the types of master regulators, the FFLs can be divided into three groups: (1) TF is master regulator, which regulates miRNA and target gene; (2) miRNA is master regulator, which regulates TF and target genes; and (3) TF and miRNA mutually regulate each other. The TF–miRNA FFL has been reported as motif that is overrepresented in regulatory network, and can minimize expression fluctuation against signaling noise [11]. During the past decade, network approach based on TF–miRNA FFLs has been demonstrated as a promising tool to dissect the etiology of many tumors [13], such as glioblastoma [14], ovarian cancer [15], non-small-cell lung cancer (NSCLC) [16] and T-cell acute lymphoblastic leukemia [17]. For example, Mitra *et al.* identified the disrupted FFLs in NSCLC from a predicted, but reproducible, TF and miRNA regulatory network, and found that miR-9-5p and miR-130b-3p could inhibit the tumor-suppressive activity of TGF-β pathway by targeting a core regulatory molecule *TGFBR2* [16]. Recently, Yan *et al.* proposed a novel method, dChip-GemiNI, to identify significant TF–miRNA FFLs altered in cancer from a computationally derived regulatory network. The authors evaluated this approach in six data sets across five tumor types, and preliminarily investigated FFLs that were cancer specific or common across multiple tumor types [18]. However, many questions about the common or specific regulatory mechanism in different tumor types have not been fully addressed, such as which FFLs are dysregulated across tumor types (pan-cancer FFLs) or in a specific cancer? What about the functions, regulatory roles and biological insights of the pan-cancer FFLs? Thus, systematic analysis of the pan-cancer TF–miRNA FFLs and their clinical applications is urgent and necessary.

The availability of large-scale RNA-seq and miRNA-seq data from The Cancer Genome Atlas (TCGA) project and the accumulation of experimentally validated TF and miRNA regulations allow us to decipher dysregulated FFLs in pan-cancer accurately. In this study, we identified the dysregulated TF–miRNA FFLs in 13 tumor types through integrating the gene and miRNA expression data of matched tumor and normal samples from TCGA into a curated TF- and miRNA-mediated regulatory network. Here, we focused on the pan-cancer FFLs to investigate their biological insights. We identified 26 pan-cancer FFLs, defined to be dysregulated in at least five tumor types. We found that they were not independent; rather, they communicated with each other to form several dense subnetworks. The genes and miRNAs in the pan-cancer FFLs showed some meaningful functional, topological and pharmacological properties, indicating that they might play important roles in tumorigenesis. In addition, targeting TFs or miRNAs is a promising strategy for molecular cancer therapy [19, 20]. Thus, we subsequently explored the application of pan-cancer FFLs in anticancer drug development. We found that the dysregulation of three FFLs, including E2F1_hsa-miR-195-5p_CCND1, hsa-miR-34a-5p_E2F1_CCND1 and JUN_hsa-miR-21-5p_MSH2, might be the potential mechanism of action (MOA) of an anticancer drug arsenic trioxide (ATO). Moreover, sulindac, which is used for the treatment of pain and fever, might have the anticancer activity. In summary, we identified the pan-cancer dysregulated FFLs, explored their function and demonstrated the potential ability to elucidate pathogenesis of cancer and develop novel anticancer drugs.

## Material and methods

### Gene and miRNA expression data across 13 tumor types

Gene and miRNA expression data in matched tumor and normal samples were obtained from TCGA project (as of September 2014). To eliminate the bias from different platforms, we only extracted gene and miRNA expression levels that were measured by Illunima HiSeq platform. As a result, we obtained the gene and miRNA expression data of 13 tumor types and matched normal samples; the sample sizes ranged from 14 to 172 (see details in Supplementary Table S1). Because we only used matched tumor and normal samples, the number of tumor samples and the number of normal samples are the same. For miRNA expression, we calculated the read counts and the reads per million values of each mature miRNA from the isoform quantification files. Next, we filtered out genes or miRNAs with low expression using the edgeR R package [21]. Only genes or miRNAs having more than one count per million (CPM) in at least half of the samples were considered as detected genes or miRNAs, and they were retained for further analysis. For example, in bladder cancer, the genes were detected if they had CPM > 1 in at least 19 (38/2) samples. Finally, the differential expression for genes and miRNAs was evaluated by edgeR based on read counts. Here, we defined the significantly differentially expressed (DE) genes and miRNAs at the threshold of false discovery rate (FDR) < 0.05 and |log_2_FC| > 1.

### The TF–miRNA regulatory network

The regulations of TFs to genes were obtained from TRANSFAC^®^ Professional database (Release: 2014.2) [7]. The regulations of miRNAs to genes were obtained from TarBase v6.0 [10] and TRANSFAC^®^ Professional database. In TarBase, we only retained the miRNA regulations that have been validated by low-throughput experiments, such as reporter gene, northern blot, western blot and quantitative PCR (qPCR). The regulations of TFs to miRNAs were obtained from TransmiR v1.2 [22] and TRANSFAC^®^ Professional database. Here, the TF and gene names were mapped to Entrez IDs, and the miRNA names were mapped to miRBase accession numbers of mature miRNAs. We constructed the background regulatory network through combining all TF and miRNA regulations, and then eliminated all self-loops. As a result, the background regulatory network consists of 10 046 regulations among 597 TFs, 498 miRNAs and 2581 target genes (Supplementary Table S2).

### Definition of the TF–miRNA FFLs

In this study, we only considered the three-node FFLs, which include a TF, a miRNA and a target protein-coding gene. According to the regulations between TF and miRNA, the FFLs can be classified into three types. In the first type of FFL, which is termed as TF-FFL, TF regulates protein-coding gene and miRNA at transcriptional level, and miRNA represses gene expression at posttranscriptional level. Similarly, the miR-FFL was defined as the structure that miRNA represses TF and gene expression, while TF regulates gene expression. In the last type of FFL, TF and miRNA mutually regulate each other to form a feed-back (FB) loop, and both of them regulate the shared target genes. Thus, we denoted it as FB-FFL.

### Identification of the dysregulated TF–miRNA FFLs in cancer

To evaluate the biological activity of a particular FFL, we integrated the differential expression of all nodes and the differential co-expression of all edges in the FFL. First, the node score was calculated through the Equation (1), which was based on the significance of differential expression [23].

(1) $$N_{i}=\varphi^{-1}\left(1-p_{i}\right)$$

where *p_i_* is the *P*-value that represents the significance of expression change determined by edgeR. *φ*^−^^1^ is the inverse normal cumulative distribution function. Next, for each edge in the FFL, we calculated the Spearman correlation coefficients based on normalized read counts in tumor samples (*r_tumor_*) and normal samples (*r_normal_*) separately. The correlations were then transformed by Fisher transformation as:

(2) $$F\left(r\right)=\frac{1}{2}\ln\frac{1+r}{1-r}$$

Here, the edge score was calculated based on the difference between correlations in tumor samples and normal samples [24] through Equations (3) and (4).

(3) $$E_{i}=\varphi^{-1}\left(1-2\times \left(1-\varphi \left(\left|D\right|\right)\right)\right)$$

(4) $$D=\frac{F\left(r_{tumor}\right)-F\left(r_{normal}\right)}{\sqrt{\frac{1.06}{n_{tumor}-3}+\frac{1.06}{n_{normal}-3}}}$$

where *n* is the number of samples. Finally, the FFL score is the weighted sum of node scores and edge scores [25, 26] as follows:

(5) $$S=\lambda \frac{\sum_{i=1}^{k}N_{i}}{\sqrt{k_{N}}}+(1-\lambda )\frac{\sum_{i=1}^{k}E_{i}}{\sqrt{k_{E}}}$$

where *k_N_* and *k_E_* are the numbers of nodes and edges in the FFL, respectively (here both of them are 3). *λ* (0 ≤ *λ* ≤ 1) is a weight parameter controlling the respective contribution of the node score and edge score. Here, we set *λ* = 0.5 to balance the contribution of nodes and edges.

We performed permutation analysis to estimate the significance of each FFL score. We first randomly selected three molecules to construct a random FFL. This process was repeated 100 000 times. Next, we calculated the FFL score for each random FFL according to the above equations and generated the null distribution of FFL scores (as shown in Supplementary Figure S1). The empirical *P*-value for an observed FFL was defined as the proportion of random FFL scores (*S_random_*) larger than the observed FFL score (*S*): *P*-value = (*N_Srandom_*_ > _*_S_*)/*N_p_*, where *N_Srandom_*_ > _*_S_* is the number of random FFLs that have larger scores than the particular FFL, and *N_p_* is the number of permutations. In this study, only FFLs with *P*-value < 0.05 were considered as dysregulated in the cancer of investigation.

### Pathway analysis of genes and miRNAs in pan-cancer FFLs

We used DAVID Bioinformatics Resources [27] to examine the enriched KEGG pathways for genes in pan-cancer FFLs. The significance level was set to FDR < 0.01. Next, we investigated the combinatorial effect on pathways of the miRNAs in pan-cancer FFLs using the DIANA miRPath Web server [28]. Here, we used ‘TarBase' to extract miRNA target genes and selected ‘pathways union' to merge the results. For each miRNA, FDR < 0.01 was used as cutoff to identify the significant pathways. Only pathways that were significantly regulated by at least five miRNAs were retained.

## Results

### Characteristics of curated TF and miRNA regulations

**Figure 1:**
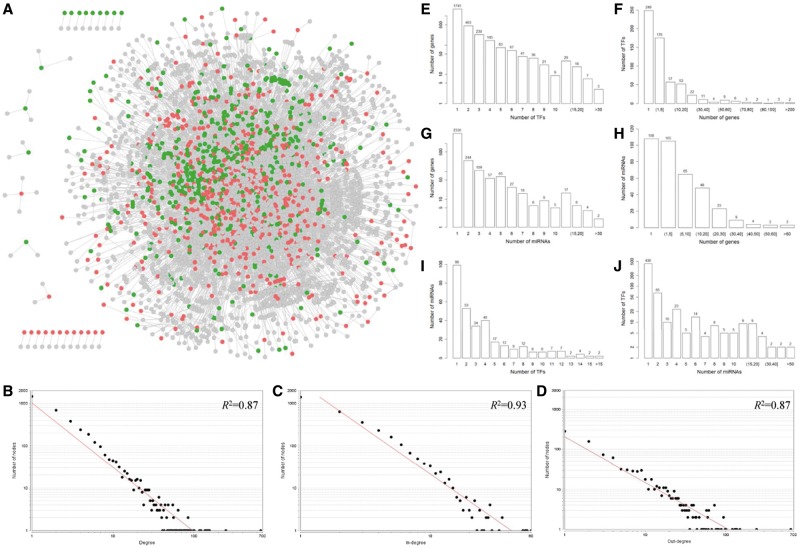
The curated TF and miRNA regulatory network and its node degree distribution. (**A**) The TF and miRNA regulatory network. Red, green and gray nodes represent TFs, miRNAs and target genes, respectively. The log-log plots show that the degree (**B**), in-degree (**C**) and out-degree (**D**) distributions follow the power law. (**E**, **F**) The relationship between the number of TFs and their target genes. (**G**, **H**) The relationship between the number of miRNAs and their target genes. (**I**, **J**) The relationship between the number of TFs and their target miRNAs. A colour version of this figure is available online at BIB online: https://academic.oup.com/bib.

We obtained the experimentally validated TF and miRNA regulations from TRANSFAC [7], TarBase [10] and TransmiR [22] databases. After eliminating all self-loops, we obtained 10 046 regulations of 597 TFs and 498 miRNAs (Supplementary Table S2). The curated TF and miRNA regulatory network was visualized in Figure 1A. To have an overview of this integrated regulatory network, we examined the degree distribution of the network by NetworkAnalyzer plug-in of Cytoscape [29]. The power law distribution of the forms *y* = 1013.8 × 10^−^^1.516^ (*R*^2 ^= 0.87), *y* = 2284.1 × 10^−^^2.127^ (*R*^2 ^= 0.93) and *y* = 199.94 × 10^−^^1.131^ (*R*^2 ^= 0.87) was fitted for degree (Figure 1B), in-degree (Figure 1C) and out-degree (Figure 1D), respectively. These results indicated that the curated TF and miRNA regulatory network satisfied approximate scale-free topology, which is the common feature of the most biological networks [30]. Next, to get an insight into the TF and miRNA regulations, we further investigated the relationship among the number of TFs, miRNAs and their targets. As shown in Figures 1E and 1F, most (92.03%) genes are regulated by a small number of TFs (i.e. ≤5 TFs), while only three genes are regulated by more than >30 TFs. On the other hand, most (71.19%) TFs regulate a few of genes (i.e. ≤5 genes), while only two TFs regulate >200 genes. Based on the prediction results of miRNA targets, a miRNA can bind several hundred genes, and a single gene can be targeted by multiple miRNAs [31]. Remarkably, in the curated regulatory network that we constructed here, >80% genes are regulated by a single miRNA (Figure 1G), and only three miRNAs have >60 validated target genes (Figure 1H). The same trend was observed for the TF regulations of miRNAs. Most (77.64%) miRNAs are regulated by only a few TFs (i.e. ≤5 TFs) (Figure 1I), and most (89.28%) TFs regulate a small number of miRNAs (i.e. ≤5 miRNAs) (Figure 1J).

### Differential expression pattern of genes and miRNAs across tumor types

**Figure 2:**
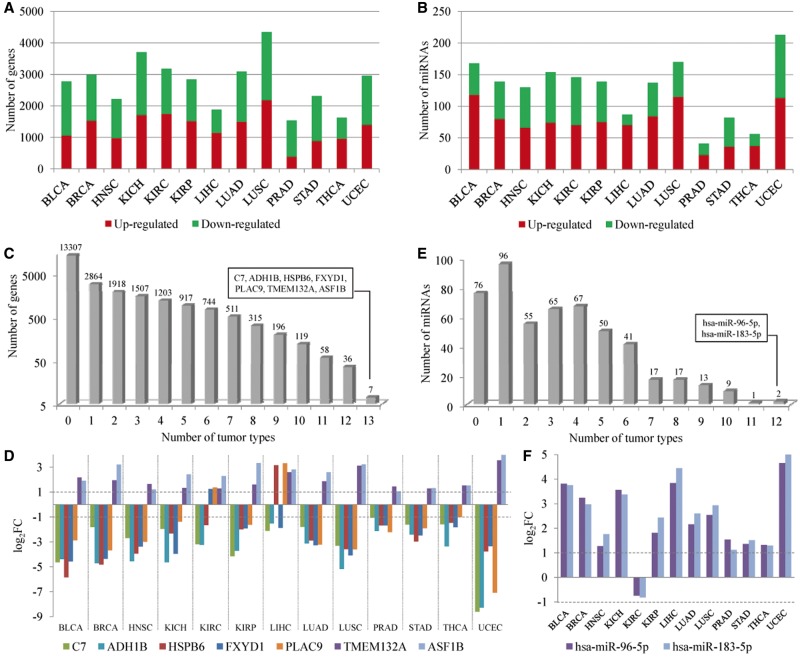
DE genes and miRNAs in 13 tumor types. (**A**) The number of upregulated (in red) and downregulated genes (in green) in 13 tumor types. (**B**) The number of upregulated (in red) and downregulated miRNAs (in green) in 13 tumor types. (**C**) The number of genes that were DE in different number of tumor types. (**D**) The expression patterns of the seven most frequently DE genes across 13 tumor types. (**E**) The number of miRNAs that were DE in different number of tumor types. (**F**) The expression patterns of the two most frequently DE miRNAs across 13 tumor types. A colour version of this figure is available online at BIB online: https://academic.oup.com/bib.

The gene and miRNA expression was measured using next-generation sequencing technologies. The data were downloaded from the TCGA (Level 3). In this study, we analyzed expression files of 13 tumor types, including bladder urothelial carcinoma (BLCA), breast invasive carcinoma (BRCA), head and neck squamous cell carcinoma (HNSC), kidney chromophobe (KICH), kidney renal clear cell carcinoma (KIRC), kidney renal papillary cell carcinoma (KIRP), liver hepatocellular carcinoma (LIHC), lung adenocarcinoma (LUAD), lung squamous cell carcinoma (LUSC), prostate adenocarcinoma (PRAD), stomach adenocarcinoma (STAD), thyroid carcinoma (THCA) and uterine corpus endometrial carcinoma (UCEC). Because the miRNAs in the curated regulatory network above are mature miRNAs, we extracted the mature miRNA expression from the isoform quantification files. Based on read counts, edgeR R package [21] was used to identify the DE genes and DE mature miRNAs. At the significance level of FDR < 0.05 and log fold change (|log_2_FC|) > 1, the number of DE genes (Figure 2A and Supplementary Table S3) and DE miRNAs (Figure 2B and Supplementary Table S4) in 13 tumor types ranged from 1536 (PRAD) to 4349 (LUSC), and from 41 (PRAD) to 213 (UCEC), respectively. Next, we investigated the relationship between the sample size and the number of DE genes or miRNAs. The results did not show significant correlations for DE genes (*P* = 0.455) or DE miRNAs (*P* = 0.139).

More than half of the genes were not DE in any tumor type, while seven genes (*C7*, *ADH1B*, *HSPB6*, *FXYD1*, *PLAC9*, *TMEM132A* and *ASF1B*) were DE in all tumor types (Figure 2C). These seven genes had consistent expression pattern across 13 tumor types (Figure 2D). For example, antisilencing function 1B histone chaperone (*ASF1B*) was upregulated in all the 13 tumor types. *ASF1B* is required for cell proliferation and is one of the most frequently overexpressed histone chaperones in cancer [32]. The higher expression of *ASF1B* may alter nucleosome assembly, resulting in genome instability and the promotion of tumorigenesis [33], and has been demonstrated to have an association with increased metastasis and poor survival of breast cancer [34]. Inhibition of *ASF1B* expression could decrease cell proliferation, indicating the potential of *ASF1B* to be a new target for treatment of cancer. In addition, *FXYD1* and *HSPB6* were overexpressed only in KIRC and LIHC, respectively, whereas they were downregulated in other types of tumor. Similarly, *PLAC9* was downregulated in most tumor types, except for KIRC and LIHC.

Furthermore, we found consistent overexpression of hsa-miR-96-5p and hsa-miR-183-5p across 12 tumor types but not in KIRC (Figure 2E and F), which was supported by the previous studies. Hsa-miR-183-5p is a potential oncogenic miRNA, and is overexpressed in prostate cancer cells and tissues. The *in vitro* and *in vivo* experiments demonstrated that inhibition of hsa-miR-183-5p could decrease prostate tumor growth [35]. This miRNA has also been reported to be upregulated in several other tumors, such as colon cancer [36], breast cancer [37], liver cancer [38] and lung cancer [39]. Concordance in previously reported dysregulation pattern of this miRNA with the results from TCGA miRNA-Seq expression profiling data suggested that our downstream analysis had been carried out using a reliable and high-quality data set.

### Dysregulated FFLs in 13 tumor types

**Figure 3:**
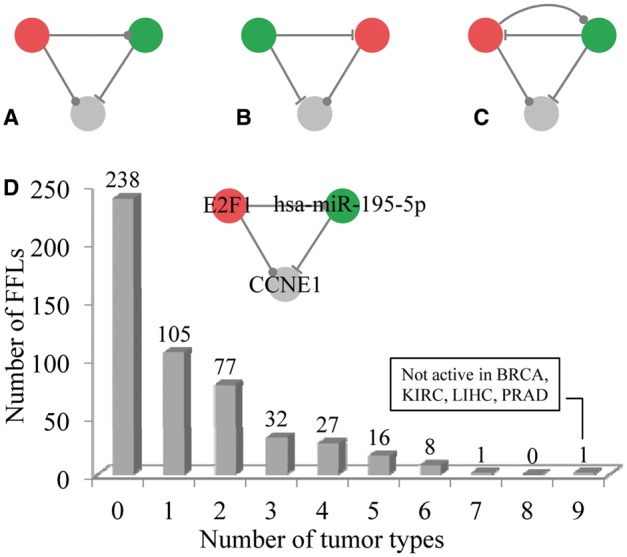
The three types of FFLs and the distribution of the number of dysregulated FFLs across tumor types. (**A**) TF-FFL: TF and miRNA regulate gene, and TF regulates miRNA. (**B**) miRNA-FFL: TF and miRNA regulate gene, and miRNA regulates TF. (**A**) FB-FFL: TF and miRNA regulate gene, and TF and miRNA mutually regulate each other. (**D**) The number of dysregulated FFLs in different number of tumor types. Specifically, E2F1_hsa-miR-195-5p_CCNE1 was dysregulated in nine tumor types except for BRCA, KIRC, LIHC and PRAD. The red, green and gray nodes represent TF, miRNA and target gene, respectively. The dots on edges indicate either transcriptional activation or inhibition, while the hammerheads denote posttranscriptional suppression. A colour version of this figure is available online at BIB online: https://academic.oup.com/bib.

Based on the regulation between TF and miRNA, there are three types of three-node FFLs, TF-mediated FFL (TF-FFL: TF and miRNA regulate gene, and TF regulates miRNA); miRNA-mediated FFL (miRNA-FFL: TF and miRNA regulate gene, and miRNA regulates TF); and FB loop-mediated FFL (FB-FFL: TF and miRNA regulate gene, and TF and miRNA mutually regulate each other) (Figure 3A–C), in the TF–miRNA regulatory network. Here, we identified 505 FFLs, including 244 TF-FFLs, 226 miRNA-FFLs and 35 FB-FFLs (Supplementary Table S5 and Supplementary Figure S2A). Through combining the changes of nodes (differential expression) and edges (differential co-expression) between tumor and normal samples, we evaluated the strength of association between each FFL and each tumor type. We defined a FFL was dysregulated in a tumor type if the score of the FFL in this tumor was significantly larger than the scores of 100 000 random FFLs (experimental *P*-value < 0.05). The significance information of all the 505 FFLs across 13 tumor types is provided in Supplementary Table S5. We identified that 267 FFLs were dysregulated in at least one tumor type (Figure 3D and Supplementary Figure S2B), among them E2F1_hsa-miR-195-5p_CCNE1 was most frequently disrupted in nine tumor types (Figure 3D). The number of significant FFLs detected in each tumor type ranged from 14 (KICH) to 94 (UCEC) (Supplementary Figure S2B). The significance of correlation between the sample size and the number of dysregulated FFLs was 0.133.

**Figure 4:**
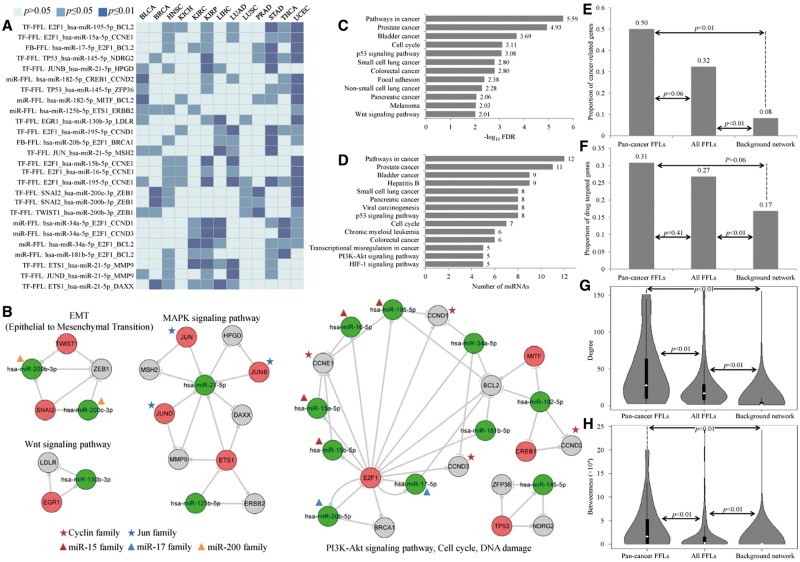
Pan-cancer FFLs and their functional analysis. (**A**) Pan-cancer FFLs and their significance in different tumor types. Light blue, blue and dark blue cubes represent the FFLs that were not significant (*P* > 0.05), significant (*P* ≤ 0.05) and strongly significant (*P* ≤ 0.01) in the corresponding tumor type. (**B**) Pan-cancer TF–miRNA regulatory network and the function of each highly connected subnetworks. The members of the cyclin and Jun family are marked with red and blue stars, respectively. Red, blue and orange triangles represent miR-15, miR-17 and miR-200 family, respectively. (**C**) The pathways that were significantly enriched with genes in pan-cancer FFLs. (**D**) The significant pathways that were affected by miRNAs in pan-cancer FFLs. (**E**) The proportion of cancer-associated genes in pan-cancer FFLs, all FFLs and background regulatory network. (**F**) The proportion of drug target genes in pan-cancer FFLs, all FFLs and background regulatory network. (**G**) The degree distribution of all nodes in pan-cancer FFLs, all FFLs and background regulatory network. (**H**) The betweenness distribution of all nodes in pan-cancer FFLs, all FFLs and background regulatory network. A colour version of this figure is available online at BIB online: https://academic.oup.com/bib.

Next, we defined the FFLs that were dysregulated in at least five tumor types as pan-cancer FFLs. This definition is arbitrary, but we thought that this category of FFLs may reflect the common regulatory mechanisms on the pathogenesis of major types of tumor. Based on this definition, we identified 26 pan-cancer FFLs, as listed in Table 1 (see details in Figure 4A and Supplementary Table S6). The number of pan-cancer FFLs (26) was significant larger than random expectation (*P* < 0.001 in 1000 permutations). Through converging the 26 FFLs, we constructed a pan-cancer TF–miRNA regulatory network, which consisted of 11 TFs, 15 miRNAs and 15 target genes (Figure 4B). In this pan-cancer regulatory network, *E2F1* acted as a hub with degree 15, indicating that it was involved in many pan-cancer FFLs. *E2F1* encodes a member of the E2F family of TFs and plays a crucial role in a wide range of cellular processes, including cell cycle, differential, apoptosis and DNA damage [40, 41]. It had also been reported that *E2F1* was deregulated in many types of cancers, such as bladder cancer, breast cancer and lung cancer [41, 42].

**Table 1:** Summary of the pan-cancer FFLs

FFL types	Dysregulated FFLs			#(tumor types)^a^
TF-FFL	E2F1	hsa-miR-195-5p	CCNE1	9
TF-FFL	E2F1	hsa-miR-195-5p	BCL2	7
TF-FFL	E2F1	hsa-miR-15a-5p	CCNE1	6
TF-FFL	E2F1	hsa-miR-195-5p	CCND1	6
TF-FFL	ETS1	hsa-miR-21-5p	MMP9	6
TF-FFL	ETS1	hsa-miR-21-5p	DAXX	6
TF-FFL	TP53	hsa-miR-145-5p	NDRG2	6
miRNA-FFL	hsa-miR-34a-5p	E2F1	CCND1	6
miRNA-FFL	hsa-miR-34a-5p	E2F1	BCL2	6
FB-FFL	hsa-miR-17-5p	E2F1	BCL2	6
TF-FFL	E2F1	hsa-miR-15b-5p	CCNE1	5
TF-FFL	E2F1	hsa-miR-16-5p	CCNE1	5
TF-FFL	EGR1	hsa-miR-130b-3p	LDLR	5
TF-FFL	JUN	hsa-miR-21-5p	MSH2	5
TF-FFL	JUNB	hsa-miR-21-5p	HPGD	5
TF-FFL	JUND	hsa-miR-21-5p	MMP9	5
TF-FFL	SNAI2	hsa-miR-200b-3p	ZEB1	5
TF-FFL	SNAI2	hsa-miR-200c-3p	ZEB1	5
TF-FFL	TP53	hsa-miR-145-5p	ZFP36	5
TF-FFL	TWIST1	hsa-miR-200b-3p	ZEB1	5
miRNA-FFL	hsa-miR-34a-5p	E2F1	CCND3	5
miRNA-FFL	hsa-miR-181b-5p	E2F1	BCL2	5
miRNA-FFL	hsa-miR-182-5p	CREB1	CCND2	5
miRNA-FFL	hsa-miR-182-5p	MITF	BCL2	5
miRNA-FFL	hsa-miR-125b-5p	ETS1	ERBB2	5
FB-FFL	hsa-miR-20b-5p	E2F1	BRCA1	5

^a^Number of tumor types that the FFL detected.

Intuitively, the pan-cancer regulatory network was composed of several independent densely connected subnetworks. Surprisingly, the molecules in these subnetworks participated in the consistent cellular processes, such as PI3K-Akt, MAPK and Wnt signaling pathways, as well as cell cycle, DNA damage and epithelial to mesenchymal transition (EMT) processes (Figure 4B). These pathways and processes were all strongly related to tumorigenesis and well documented in literature. For example, EMT is a process by which cells lose their epithelial characteristics and acquire properties of mesenchymal cells and the ability to migrate, which usually occurs in the initiation of metastasis for cancer progression [43]. In the EMT-related subnetwork (the upper left subnetwork in Figure 4B), TWIST1, SNAI2 and ZEB1 had been proved as key inducers of EMT, and had demonstrated overexpression in many cancers [44]. In addition, the miR-200 family members, such as miR-200b and miR-200c, act as new epithelial markers and repressors of EMT through inhibiting their target genes *ZEB1* and *ZEB2* [45]. Thus, this subnetwork had been validated as the key regulatory module for EMT.

To systematically identify pathways associated with the pan-cancer FFLs, we used DAVID Bioinformatics Resources [27] to identify KEGG pathways that were enriched with genes in pan-cancer FFLs. At the significance level of FDR < 0.01, we found that most of the pathways were cancer-specific pathways, such as prostate cancer pathway and bladder cancer pathway, as well as P53 signaling, focal adhesion and Wnt signaling pathways (Figure 4C). To further evaluate the combinatorial effect of the 15 miRNAs in pan-cancer FFLs on KEGG pathways, we used the DIANA miRPath Web server [28] to perform the pathway analysis[46]. Fourteen pathways were found to be significantly regulated by at least five miRNAs (FDR < 0.01). Similar to the result of genes in pan-cancer FFLs, most of the 14 pathways were cancer specific, as well as some well-known cancer-related pathways, such as P53 signaling pathway, cell cycle and PI3K-Akt signaling pathway (Figure 4D).

We found some highly connected protein families or miRNA families, and their regulations. As mentioned above, two members of miR-200 family, hsa-miR-200b-3p and hsa-miR-200c-3p were involved in the EMT process and inhibited *ZEB1* expression. Hsa-miR-17-5p and hsa-miR-20b-5p belonged to miR-17 family. Both of them formed FB loop with E2F1. Four miR-15 family members, involving hsa-miR-15a, hsa-mir-15b, hsa- miR-16 and hsa-miR-195, were direct targets of E2F1. JUN, JUNB and JUND are members of JUN protein family, which can control cell cycle, proliferation and differentiation processes, and contribute to malignant transformation [47]. In the pan-cancer FFL subnetwork, all JUN family members regulated the expression of hsa-miR-21-5p. Finally, four members of cyclin family, CCND1, CCND2, CCND3 and CCNE1, were involved in the same subnetwork, in which CCND1, CCND3 and CCNE1 were also regulated by the same TF, E2F1. Cyclin family had been demonstrated to be overexpressed in many cancers, and had been proved to be oncogenic and contributing to the pathogenesis of cancer [48]. By extracting all pan-cancer FFLs that involve cyclin family members (Supplementary Figure S2C), we found dense regulations between miR-15 family and cyclin family, and all of them were targets of E2F1 (Supplementary Figure S2D), indicating that E2F1 was a pan-cancer TF, and achieved its function through FFLs with the miR-15 family and the cyclin family.

We further analyzed the pan-cancer FFLs from multiple functional and topological properties. First, we obtained the cancer-associated genes from the Cancer Gene Census database, which deposits and carefully curates the mutated genes that are causally implicated in cancer [49]. We found that 13 (50%) genes in pan-cancer FFLs were cancer-associated genes, marginally significantly more than that in all the FFLs (32%, Fisher's exact test *P*-value = 0.06) and significantly more than that in the whole background regulatory network (8%, Fisher's exact test *P*-value < 0.01). Meanwhile, the proportion of cancer-associated genes in all FFLs was significantly larger than background network (Fisher's exact test *P*-value < 0.01). The result indicated that genes in pan-cancer FFLs were preferred to be cancer-associated genes (Figure 4E). Second, we downloaded all the targets of the US Food and Drug Administration (FDA)-approved drugs from the DrugBank database [50]. We found that the proportion of drug targets in pan-cancer FFLs (31%) and all FFLs (27%) were significantly larger than that in the background network (17%) (Fisher's exact test *P*-value < 0.01). In addition, the proportion of drug targets in pan-cancer FFLs was more than all FFLs (Figure 4F), although the difference was not significant. The result suggested that the gene products in pan-cancer FFLs were more likely to be druggable. Finally, we calculated two widely used topological properties, degree and betweenness, to investigate the important roles of pan-cancer FFLs in the regulatory network. Nodes with high degree are highly connected and referred to hubs [30, 51], while nodes with high betweenness control the fraction of information flow and are referred to bottlenecks [52]. We found that the nodes in the pan-cancer FFLs had significant higher degree (Figure 4G) and betweenness (Figure 4H) than that in all FFLs and background network (Wilcoxon rank sum test *P*-value < 0.01). This comparison indicated that nodes in pan-cancer FFLs tended to be the hubs and bottlenecks in the curated TF–miRNA regulatory network, implying their important functional roles.

From the identified dysregulated FFLs in different tumor types (Supplementary Table S5), we could also find the cancer-specific FFLs. There were 105 FFLs that were only disrupted in one specific tumor type (Figure 3D). For example, IRF1_hsa- miR-155-5p_VCAM1 was found to be dysregulated only in KIRC. We found that all of the three molecules were significantly DE in KIRC. The *P*-values of IRF1, hsa-miR-155-5p and *VCAM1* were 1.12 × 10^−^^18^, 2.64 × 10^−^^35^ and 1.34 × 10^−^^82^, respectively. In addition, the dysregulated FFLs could be used to identify the common FFLs shared by some tumor types of interest. For example, lung adenocarcinoma and lung squamous cell carcinoma are two main subtypes of NSCLC. We found that there were five common FFLs altered in both subtypes of NSCLC, involving E2F1_hsa-miR-195-5p_CCNE1, E2F1_hsa-let-7a-5p_AURKB, E2F1_hsa-miR-92a-3p_BCL2L11, EGR1_hsa-miR-30a-5p_GNAQ and SPI1_hsa-miR-146a-5p_TLR4. Most of the genes and miRNAs in these five FFLs had been examined and are aberrantly expressed in NSCLC [53].

### Potential MOA of ATO and drug repositioning

**Figure 5:**
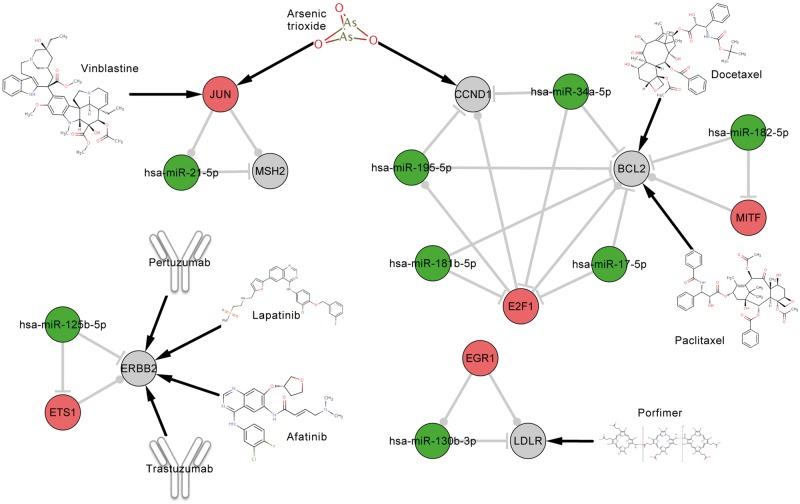
The pan-cancer FFLs that were targeted by nine anticancer drugs. The red, green and gray nodes represent TF, miRNA and target gene, respectively.

**Figure 6:**
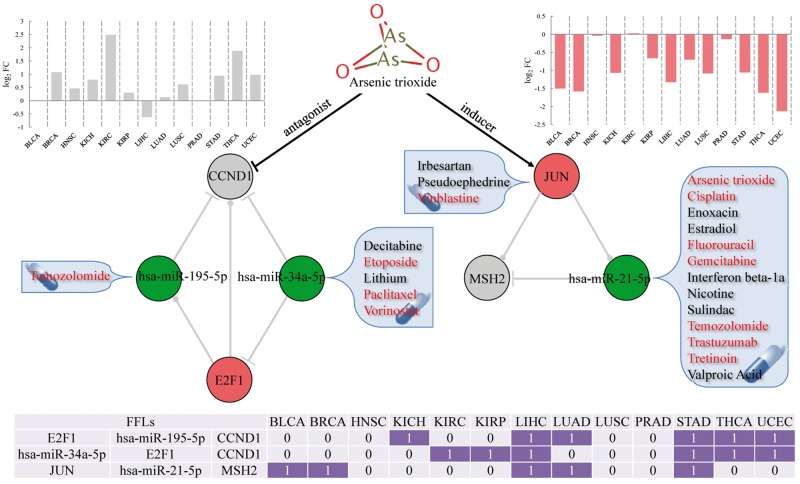
The pan-cancer FFLs that were affected by arsenic trioxide. The bar plot in gray represents the expression pattern of *CCND1* across 13 tumor types. The bar plot in red represents the expression pattern of *JUN* across 13 tumor types. The drugs in red are anticancer drugs. The bottom table summarizes the dysregulation of the FFL in the corresponding cancer; 1 denotes dysregulation, while 0 denotes no dysregulation. A colour version of this figure is available online at BIB online: https://academic.oup.com/bib.

In the above analyses, we found that the pan-cancer FFLs contained the highest proportion of drug target genes compared with all the FFLs and the whole regulatory network, indicating that gene products in pan-cancer FFLs were favored to be druggable. Here, we further analyzed the anticancer drugs that targeted the gene products in pan-cancer FFLs. First, we obtained the anticancer drugs according to the Anatomical Therapeutic Chemical (ATC) classification system. If one drug has the first two levels of ATC code L01 (antineoplastic agents), we considered this drug as anticancer drug. We found a total of 21 FDA-approved drugs that could target gene products in pan-cancer FFLs, among which 9 drugs had the ATC code L01 (Table 2). By comparing the proportion of all drugs with the L01 ATC code, we found that the gene products in pan-cancer FFLs were more likely to be targeted by anticancer drugs (hypergeometric test *P*-value = 1.48 × 10^−^^6^). Second, we constructed the pan-cancer FFLs subnetworks that were targeted by the nine anticancer drugs (Figure 5), in which ATO has two drug targets, CCND1 and JUN. ATO is an ancient drug used in both traditional Chinese and Western medicine, and has been demonstrated to have anticancer activity, especially in acute promyelocytic leukemia through promoting degradation of PML-RARα Oncoprotein [54, 55]. ATO can induce apoptosis, inhibit angiogenesis and promote differentiation [56, 57], and thus, it has been investigated as one promising general anticancer drug for both hematologic cancer and solid tumors [56–58], such as esophageal cancer [57], neuroblastoma [57], lymphoma [59], hepatocellular carcinoma [60] and sarcoma [61]. However, ATO can cause serious and sometimes fatal side effects because of the toxic nature of arsenic. The MOA of ATO is currently not completely understood. In the identified pan-cancer FFLs, CCND1 and JUN are drug targets of ATO, which span three pan-cancer FFLs (Figure 6). ATO is an antagonist of CCND1, which has been validated in many cancers, such as bladder cancer [62], breast cancer [63], kidney cancer [64], liver cancer [65] and NSCLC [66]. In this study, we found that *CCND1* was upregulated in most of the tumor types (Figure 6). Meanwhile, ATO has been demonstrated to be an inducer of JUN through regulating JNK and MAPK pathways in many cancers, such as bladder cancer [67], prostate cancer [68], NSCLC [69], liver cancer [70] and endometrial cancer [71]. Here, *JUN* was downregulated in almost all tumor types in our pan-cancer analysis (Figure 6). These results might suggest a novel potential MOA that the anticancer activity of ATO was generated through regulation of CCND1 and JUN, which in turn affected the TF–miRNA regulatory FFLs, involving E2F1_hsa-miR-195-5p_CCND1, hsa-miR-34a-5p_E2F1_CCND1 and JUN_hsa-miR-21-5p_MSH2. Finally, based on the three pan-cancer FFLs that were affected by ATO, we could predict the repurposing drugs for cancer therapy. For example, three other drugs, irbesartan, pseudoephedrine and vinblastine, can target JUN (Figure 6), indicating that the three drugs may affect the pan-cancer FFL JUN_hsa-miR-21-5p_MSH2 through mediating their drug target JUN as the mechanism of ATO. In addition, we obtained the drugs that can affect miRNA expression from the SM2miR database, which collects the experimentally validated small molecule drug effects on miRNA expression [72]. We found that the expression of hsa-miR-195-5p, hsa-miR-34a-5p and hsa-miR-21-5p could be regulated by some other FDA-approved drugs, suggesting that they might have the anticancer activity through modulating the pan-cancer FFLs. Interestingly, most of the repurposing candidates are known anticancer drugs (marked with red color in Figure 6), such as vinblastine, etoposide and paclitaxel. This demonstrated the reliability of our approach. Thus, the other drugs were more likely to have anticancer activity. For example, sulindac inhibits the expression of hsa-miR-21-5p [73] and might affect the pan-cancer FFL JUN_hsa-miR-21-5p_MSH2 (Figure 6). Sulindac is one of the nonsteroidal anti-inflammatory drugs, which has been shown to inhibit the growth of many kinds of cancer, including colon, esophagus, stomach, skin, breast, lung, prostate and urinary bladder [74]. Sulindac, which is commonly prescribed for the treatment of pain and fever, and to help ease arthritis symptoms, has been demonstrated as a potential anticancer treatment that induces apoptosis by binding to RXR-alpha [75].

**Table 2:** FDA-approved drugs that targeted genes in the pan-cancer FFLs

Drug	Target	ATC code
Afatinib	ERBB2	Antineoplastic agents (L01)
Arsenic trioxide	CCND1, JUN	Antineoplastic agents (L01)
Docetaxel	BCL2	Antineoplastic agents (L01)
Lapatinib	ERBB2	Antineoplastic agents (L01)
Paclitaxel	BCL2	Antineoplastic agents (L01)
Pertuzumab	ERBB2	Antineoplastic agents (L01)
Porfimer	LDLR	Antineoplastic agents (L01)
Trastuzumab	ERBB2	Antineoplastic agents (L01)
Vinblastine	JUN	Antineoplastic agents (L01)
Acetylsalicylic acid	TP53	Stomatological preparations (A01)
		Antithrombotic agents (B01)
		Analgesics (N02)
Minocycline	MMP9	Stomatological preparations (A01)
		Antibacterials for systemic use (J01)
Ibuprofen	BCL2	Cardiac therapy (C01)
		Other gynecologicals (G02)
		Anti-inflammatory and antirheumatic products (M01)
		Topical products for joint and muscular pain (M02)
Captopril	MMP9	Agents acting on the renin-angiotensin system (C09)
Irbesartan	JUN	Agents acting on the renin-angiotensin system (C09)
Glucosamine	MMP9	Anti-inflammatory and antirheumatic products (M01)
Rasagiline	BCL2	Antiparkinson drugs (N04)
Pseudoephedrine	JUN	Nasal preparations (R01)
Naloxone	CREB1	All other therapeutic products (V03)
Adenosine monophosphate	CREB1	NA
Ado-trastuzumab emtansine	ERBB2	NA
Marimastat	MMP9	NA

## Discussion

TFs and miRNAs are key regulators of gene expression at the transcriptional and posttranscriptional level, respectively. Many lines of evidence support that they mediate the target gene expression through coordinated regulation, and form the FFLs, which are significantly overrepresented in the mammalian regulatory network [11, 76]. TF–miRNA FFLs influence many biological processes mainly as noise buffering [13]. Over the past decade, TF–miRNA FFLs have been widely used to identify the cancer-associated genes or miRNAs in many tumor types, such as NSCLC [16], glioblastoma [14], ovarian cancer [15] and T-cell acute lymphoblastic leukemia [17]. However, such studies have focused on the individual tumor type. Some critical questions have not been investigated, such as (1) are there common FFLs that are dysregulated in multiple tumor types? (2) Do these common FFLs provide an alternative way for understanding tumorigenesis and drug development? Here, we performed a comprehensive analysis of TF–miRNA FFLs across 13 tumor types to identify the pan-cancer FFLs, investigate the biological meanings and explore the potential clinical application of the pan-cancer FFLs.

In this study, we first constructed the human TF–miRNA regulatory network based on experimentally validated TF and miRNA regulations. In the procedure of construction of background regulatory network, we did not use the cancer-specific gene or miRNA expression data. Thus, the network we constructed in this study was general and cancer independent. In the following analysis, through incorporating the expression profiles, we could identify disrupted FFLs in each tumor type. To make the analysis more reliable, we extracted the gene and miRNA expression in matched tumor and normal samples for 13 tumor types from TCGA. Next, we identified the DE genes and miRNAs for each tumor type, which can be used to discover the pan-cancer DE genes and miRNAs. Because many ‘master regulators' of a disease process cannot be captured by differential expression analysis, differential co-expression analysis has been developed and widely used for identification of disease genes in the context of the regulatory systems [77]. The differential co-expression approach is complementary to differential expression analysis, which can not only capture dynamic behaviors of disease genes, but also identify genes with moderate differential expression signals. Here, we calculated the association strength of one FFL with one tumor type by combining the differential expression of nodes and differential co-expression of edges. We identified 26 pan-cancer FFLs that were significantly dysregulated in at least five tumor types, in which there were 18 TF-FFLs, 7 miR-FFLs and 1 FB-FFL. Using hypergeometric tests, we found that pan-cancer FFLs significantly enriched only with TF-FFLs (*P* = 0.023), which indicated that TFs acted as master regulators and might play more important roles in tumorigenesis. In addition, we obtained one newly released gene and miRNA expression data in 11 esophageal carcinoma (ESCA) samples and 11 match normal samples from TCGA as an independent data set. Of the 26 pan-cancer FFLs, 11 FFLs were significant in ESCA. The hypergeometric test showed that the dysregulated FFLs were significantly enriched with pan-cancer FFLs (*P* = 0.006), which indicated that the pan-cancer FFLs were reproducible to some extent. Furthermore, the sample size of the 13 tumor types ranged from UCEC (*n* = 14) to BRCA (*n* = 172). This variation might affect the statistical tests and the stability of our results. Thus, we randomly selected a portion of samples to evaluate this affection. For example, BRCA had 86 tumor samples and 86 matched normal samples. We randomly selected 60, 40 and 20 pairs of tumor and normal samples to perform the same analysis. The results showed that the ranks of differential expression of genes and miRNAs, as well as the significance of FFLs in different sample sizes, were all significantly correlated by Spearman correlation, which also indicated that our results were stable and reproducible.

To our knowledge, this study represents the first one to identify dysregulated FFLs across multiple tumor types based on experimentally validated TF and miRNA regulations. Thus, we explored the biological insights of the pan-cancer FFLs in depth, including both the viewpoints of function and network topology. The identified pan-cancer FFLs formed several densely connected subnetworks. Most of the genes and miRNAs in each subnetwork participated in specific cellular processes and are well documented to have important roles in tumorigenesis. Next, the pathway enrichment analysis revealed that the pan-cancer FFLs were strongly associated with cancer-related pathways, such as P53, Wnt and PI3K-Akt signaling pathways, as well as focal adhesion and cell cycle processes. In addition, we found that the genes in pan-cancer FFLs favored to be cancer-associated genes and drug targets, which indicated that these genes might have the potential to be drug targets with good druggable properties. Thus, targeting the genes in pan-cancer FFLs might be a novel cancer therapeutic. Furthermore, we investigated the relationship between these genes and patient outcomes by survival analysis using PROGgeneV2 tool [78]. The results showed that most of the genes were significantly related to prognosis in different tumor types, which indicated that these genes had the clinical significance. Finally, the topological analysis revealed that the molecules in pan-cancer FFLs were more likely to be the hubs and bottlenecks. Collectively, all these results indicated that the pan-cancer FFLs were not only densely connected in terms of network topology but also highly associated with cancer diagnosis, prognosis and treatment.

In this study, we further explored the clinical application of pan-cancer FFLs for drug development. We found that the gene products in pan-cancer FFLs tended to be targeted by anticancer drugs. The FFLs may act as functional modules, providing the potential MOA of drugs. In the pan-cancer FFLs, two genes, *CCND1* and *JUN*, are drug targets of ATO. *CCND1* was overexpressed in most of the tumor types. ATO is the antagonist of *CCND1*, which means it can inhibit the expression of *CCND1*. In addition, ATO is the inducer of *JUN*, which was frequently downregulated in almost all tumor types. There were three TF–miRNA FFLs that involved *CCND1* and *JUN*. The disruption of the three FFLs might be the potential MOA of ATO. Furthermore, we also found several FDA-approved drugs that can target or affect the other genes or miRNAs in the three FFLs. Interestingly, most of the drugs are anticancer drugs, indicating that the pan-cancer FFLs might be used for drug repositioning. For example, we revealed that sulindac, a prescription drug for the treatment of pain and fever, might have potential anticancer activity.

In summary, we identified the TF–miRNA FFLs in a curated regulatory network, the DE genes and the miRNAs between tumor and matched normal samples for 13 tumor types, and the pan-cancer FFLs in the present study. Our analysis revealed the potential worth of pan-cancer FFLs in uncovering of the pathogenesis of cancer and the MOA of drugs, as well as the drug repositioning.

Key Points

- We have proposed an integrative systems biology approach to identify dysregulated feed-forward loops across multiple tumor types (pan-cancer FFLs).
- Cancer-associated genes and drug targets were enriched in the pan-cancer FFLs.
- The genes and miRNAs in pan-cancer FFLs tended to be network hubs and bottlenecks.
- The pan-cancer FFLs had potential to predict anticancer indications for existing drugs with novel mechanism of action.
- The dysregulation of three FFLs, including E2F1_hsa-miR-195-5p_CCND1, hsa-miR-34a-5p_E2F1_CCND1 and JUN_hsa-miR-21-5p_MSH2, might be the potential MOA of an anticancer drug arsenic trioxide.

## Supplementary Data

Supplementary data are available online at https://academic.oup.com/bib.

## Funding

This work was supported by the National Institutes of Health (NIH) grants (R01LM011177, R03CA167695, P30CA68485, P50CA095103, and P50CA090949), the Robert J. Kleberg, Jr. and Helen C. Kleberg Foundation (to Z.Z.) and the Ingram Professorship Funds (to ZZ). The funders had no role in study design, data collection and analysis, decision to publish or preparation of the manuscript.

## Supplementary Material

Supplementary Data
