# Supplementary material for: Systematic dissection of dysregulated transcription factor–miRNA feed-forward loops across tumor types
Source: Brief Bioinform. 2015 Dec 9;17(6):996–1008. doi: 10.1093/bib/bbv107 (PMC5142013; doi:10.1093/bib/bbv107)
Supplement: Supplementary Data [file supp_17_6_996__index.html]

Systematic dissection of dysregulated transcription factor–miRNA feed-forward loops across tumor types — Systematic dissection of dysregulated transcription factor–miRNA feed-forward loops across tumor types — Supplementary Data 

# Systematic dissection of dysregulated transcription factor–miRNA feed-forward loops across tumor types

## Supplementary Data

files

- Supplementary Data - zip file
