# Supplementary figures and images for: Systematic dissection of dysregulated transcription factor–miRNA feed-forward loops across tumor types
Source: Brief Bioinform. 2015 Dec 9;17(6):996–1008. doi: 10.1093/bib/bbv107 (PMC5142013; doi:10.1093/bib/bbv107)

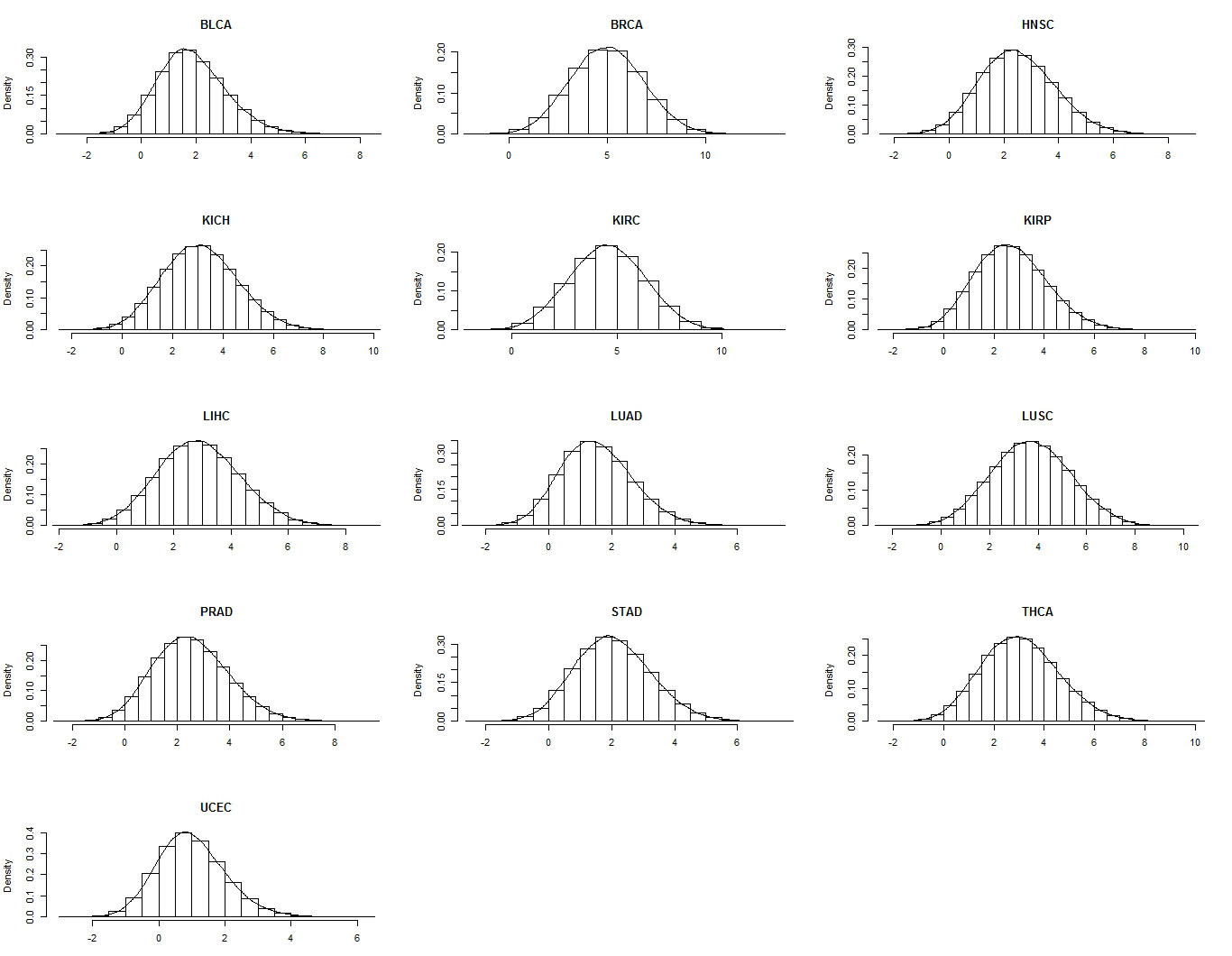

Supplement: Supplementary Data [file supp_bbv107_suppl_data.zip › Figure S1.jpg]

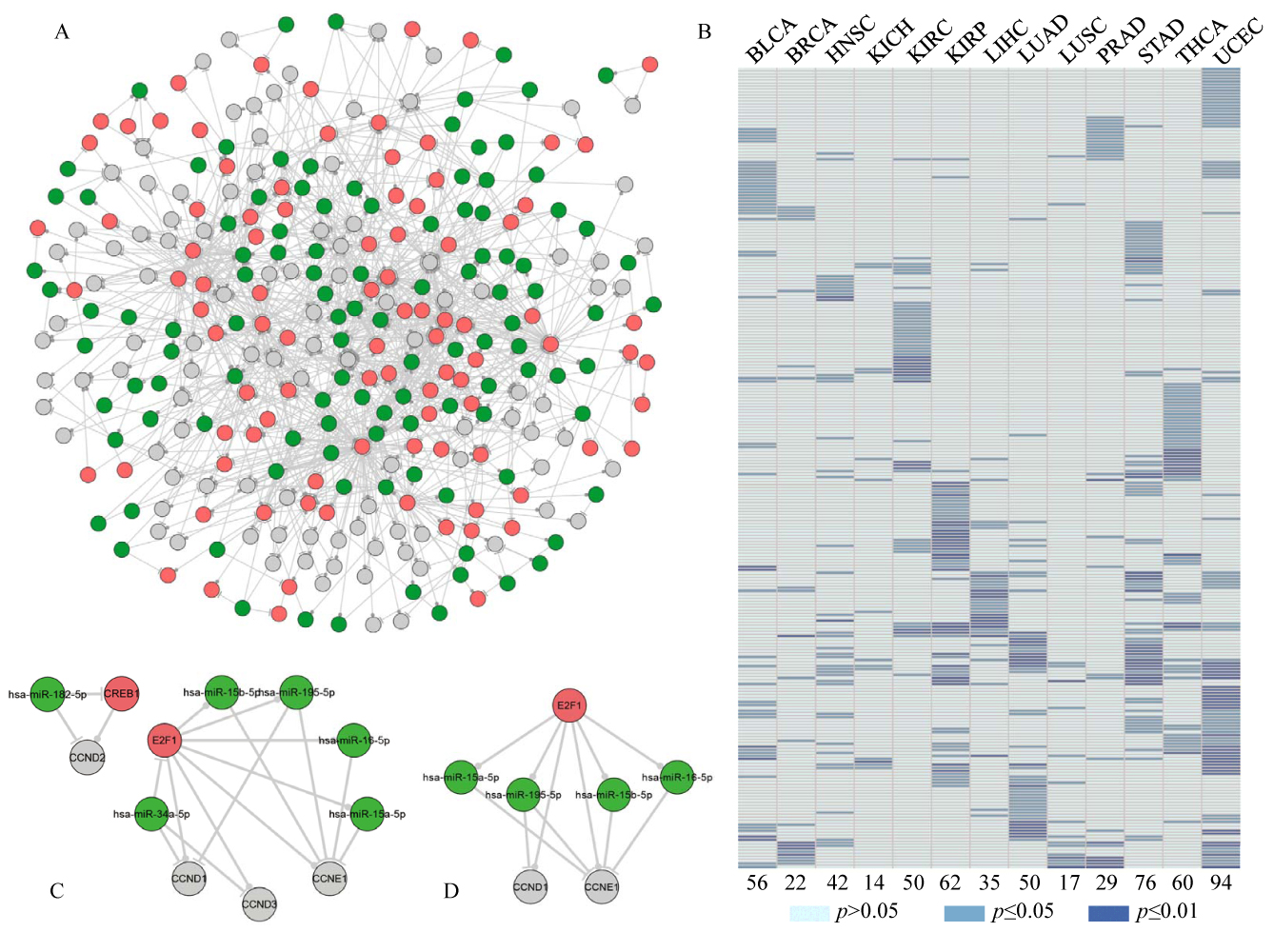

Supplement: Supplementary Data [file supp_bbv107_suppl_data.zip › Figure S2.jpg]
